# Cancer genetics services: a systematic review of the economic evidence and issues

**DOI:** 10.1038/sj.bjc.6601792

**Published:** 2004-04-13

**Authors:** G L Griffith, R T Edwards, J Gray

**Affiliations:** 1 Centre for the Economics of Health, Institute of Medical and Social Care Research, University of Wales, Wheldon Building, Bangor, Gwynedd LL57 2UW, UK; 2 Institute of Medical Genetics, Cardiff and Vale NHS Trust, University Hospital of Wales, Cardiff CF14 4XW, UK

**Keywords:** genetic, familial, cancer, systematic review

## Abstract

This paper systematically reviews the published economic research upon cancer genetics services for families at risk of having familial breast, ovarian or colorectal cancer. A structured search was made of 15 electronic databases. The search identified 1030 papers, of which 31 fulfilled the inclusion criteria, two were cost–benefit studies, five were cost consequences, four were cost-effectiveness studies, one was a cost analysis, two were cost-minimisation studies, one was a cost–utility study, 10 modelled life years and six were reviews. Modelling studies indicate that surveillance, prophylactic and chemoprevention techniques extend survival for carriers of identified mutations. Genetic testing has been estimated to cost $70–2400 [£48^*^–1591] and genetic counselling $129–800 [£89–£551]. The technology of genetic testing has been found to be cost effective. Cost effectiveness was particularly influenced by targeting genetic services for patients with a strong family history of cancer rather than screening the entire population. Future economic evaluation must go beyond merely assessing health outcomes and mutation identification, and account for the impact of genetic services upon the individual, the family and society, establish the value of services to these groups and determine the most effective ways of delivering genetic services.

It is estimated that 5–10% of breast cancer cases (King *et al*, 1993; Eeles, 1996), 10% of ovarian cancer cases (Easton *et al*, 1993) and 10–15% of colorectal cancer cases are due to inherited genetic mutations (Soravia *et al*, 1997). Women with a BRCA1 mutation have a lifetime risk of up to 80–90% of developing breast cancer, 40–60% chance of developing ovarian cancer and possibly an increased risk of developing colorectal cancer (Ford *et al*, 1994). Mutations in the hereditary genes nonpolyposis colorectal cancer (HNPCC) and familial adenomatous polyposis (FAP) predispose carriers particularly to colorectal cancer. A HNPCC germline mutation conferes up to an 80–90% lifetime risk of developing colorectal cancer (Vasen *et al*, 1996, 1998; Soravia *et al*, 1997). Carriers of a FAP mutation have an 80–100% chance of developing colorectal cancer during their lifetime (Haggitt and Reid, 1986). Hereditary genes nonpolyposis colorectal cancer and FAP also convey risks of developing cancers in the duodenum, ureteric tract and other organs (Vasen *et al*, 1991; Soravia *et al*, 1997).

As a consequence of increased awareness among the general public, there has been demand for genetic assessment services (Priority Areas Cancer Team, 1998; Ponder, 1999). However, media speculation suggests that, as a result of the revolution in genetics, the NHS will become too expensive to run (Sylvester, 2000), posing the question: what do we currently know about the provision of cancer genetic services? In this paper, we review the evidence and issues highlighted in economic evaluations of cancer genetic services for breast, ovarian and colorectal cancer.

## MATERIALS AND METHODS

### Search strategy

A structured search was conducted to identify evidence relating to the economic evaluation of cancer genetic services for families at risk of having familial breast, ovarian or colorectal cancer. This was done by means of searching the electronic databases: BMJ Archive, BIDS, Medline, HealthPromis, DARE, EED, HTA, Cambridge Scientific Abstracts, Econobase, CINAHL, ASSIA, British Library Catalogue, OCULC WorldCat, Resource Discovery Network and the Cochran Library. Titles, abstracts and articles were searched for the terms cancer, genetic and economic or cost. Retrieved papers were hand searched for references that had not been identified in the electronic search.

### Inclusion and exclusion criteria

The criteria for including literature in this paper was as follows:

1. a general review article;
2. a partial or full economic evaluation of treatment;
3. a partial or full economic evaluation of detection or counselling of individuals for familial cancer.

Research was excluded from the review if it:

1. was published in any language other than English;
2. examined sporadic cancer;
3. examined a form of genetic cancer other than breast, ovarian or colorectal cancer such as haemochromatosis;
4. was not an economics paper and only alluded to potential economic savings;
5. if the term cost had been used to refer to the potentially negative psychosocial consequences of cancer genetic services.

### Data extraction and analysis

All abstracts were obtained and assessed for relevance. All articles found to be relevant or potentially relevant were obtained and reviewed in full by one economist to ensure consistency. For all the empirical papers included in the review, the elements identified in Drummond *et al*'s (1997) 10-item checklist for a sound economic evaluation (see Table 1

**Table 1:** Drummond *et al*'s (1997) 10-item checklist for a sound economic evaluation

1	Was a well-defined question posed in an answerable form?
2	Was a comprehensive description of the competing alternatives given?
3	Was the effectiveness of the programmes or services established?
4	Were all the important and relevant costs and consequences for each alternative identified?
5	Were costs and consequences measured accurately in appropriate physical units?
6	Were costs and consequences valued credibly?
7	Were costs and consequences adjusted for differential timing?
8	Was an incremental analysis of costs and consequences of alternatives performed?
9	Was allowance made for uncertainty in the establishments of costs and consequences?
10	Did the presentation and discussion of study results include all issues of concern to users?

) were sought. These elements were then appraised, as were their inter-relationships. Meta-analysis was not possible due to the diversity in research methods, genetic testing and interventions used, and reliance on estimated health outcomes due to a lack of trial, cancer registry and observational data.

For comparative purposes, we have converted all cost estimates to 2002/3 £ and placed them in square brackets. Exchange rates were taken from the X-rates.com website, while NHS costs estimates were inflated using the Hospital and Community Pay and Price Index (Netten and Curtis, 2003). All other costs estimates were inflated using the HM Treasury GDP Deflator (2003). Authors who had not identified the year for which their costs were estimated were contacted and asked to provide this information. Five authors did not respond. For these five papers, the financial year was estimated by subtracting 2 years from the date of publication; these estimates are identified by an ^*^ following the estimate.

## RESULTS

### Included studies

The search identified 1030 papers, of which 31 fulfilled the inclusion criteria. Of the 31 papers covering 29 studies, two were cost–benefit studies, five were cost consequences, four were cost-effectiveness studies, one was a cost analysis, two were cost-minimization studies, one was a cost–utility study, 10 modelled life years (five also considered costs) and six were reviews. The 31 studies are listed in Table 2

**Table 2:** Form of evaluation

**Name**	**Year**	**CB**	**CC**	**CE**	**CA**	**CM**	**CU**	**MLY**	**R**	**D10**
Maher *et al*	1993	P								4/10
Chaliki *et al*	1995	P (WTP)								1/3
Brown and Kessler	1995, 1996		P							4/10
Heimdal *et al*	1999		F							7/9
(TRACE)			F							9/9
Brain *et al*	2000									
Cohen *et al*	In press									
Eccles *et al*	1998			F						5/9
Lidereau *et al*	2000			F						5/9
Debniak *et al*	2000			F						5/9
Sevilla *et al*	2002			F						8/9
Van Orsouw *et al*	1999				P					3/9
Cromwell *et al*	1998					P				7/10
Bapat *et al*	1999					P				9/10
Wilson *et al*	1999						F			8/9
Vasen *et al*	1998		✓					F		8/10
Syngal *et al*	1998							P		7/9
Schrag *et al*	1997							P		6/9
Schrag *et al*	2000							P		7/9
Grann *et al*	1998		✓					F		6/10
Grann *et al*	1999			✓				F		7/10
Grann *et al*	2000		✓					F		7/10
Grann *et al*	2002							P		7/9
Tengs *et al*	1998							P		7/9
Tengs and Berry	2000			✓				F		8/10
Lerman	1997								✓	NA
Peters and Biesecker	1997								✓	NA
Priority Areas Cancer Team	1998								✓	NA
Hall *et al*	1998								✓	NA
Steel *et al*	1999								✓	NA
Edwards	2001								✓	NA

CB=cost–benefit analysis, CC=cost-consequences analysis, CE=cost-effectiveness analysis, CA=cost analysis, CM=cost minimisation, CU=cost–utility analysis, MLE=modelling life expectancy, R=reviews or survey, WTP=willingness to pay, D10=proportion of Drummond *et al*'s (1997) 10-item checklist that were fulfilled. P indicates a partial economic evaluation and F a full economic evaluation.

and summarised in an Supplementary Appendix (available at http://www.bangor.ac.uk/health economics/Text/cancer/litappen dix) according to the form of economic evaluation used in the studies; at times the classification employed differs from those of the original authors, for example, cost effectiveness using more than one outcome variable has been classified as cost-consequences analysis.

### Quality assessment

The majority of discrepancies identified in papers related to a lack of detail provided upon costs and methods. Of the 25 empirical papers, 15 provided insufficient detail to determine whether or not they had identified all relevant costs and consequences. A total of 13 papers provided inadequate detail upon the measurement of costs and consequences. In all, 12 papers were vague or failed to value costs and consequences credibly. Seven papers did not discount costs and/or consequences for differential timing. A total of 14 papers neglected to identify the year for which their costs were estimated and six papers did not adequately conduct sensitivity analysis to account for uncertainty in the estimates of costs and consequences.

## DISCUSSION

The body of research conducted over the past decade into cancer genetic services and technology has been seeking to answer the key questions: What are the outcomes or the advantages and disadvantages of cancer genetic testing and services? What are the financial costs of conducting testing? What value do patients place upon genetic services? What is the most efficient method of providing genetic services?

### Outcomes of genetic services

#### Measures

To date, health economics research assessing the advantages and disadvantages of cancer genetics services has concentrated upon health outcomes such as the number of cancers detected, the number of mutations detected and, in particular, modelling survival and quality-adjusted survival (QALYs), acknowledging death and the impact of cancer upon quality of life as the disadvantages, and diagnosis and survival as the benefits of genetic services. Exceptions to this were the TRACE project (Brain *et al*, 2000; Cohen *et al*, in press), utilising a range of psychosocial measures including reasons for attendance at a genetics clinic, and the work of Wilson *et al* (1999) assessing the utility of genetic counselling to past users in terms of process attributes. Similar findings were reported by Hall *et al* (1998) in their non-systematic review of genetic testing, in which they report that evaluations of genetic diseases have confined themselves to positive health effects and those utilising a social perspective to the total effects on individuals. For example, while outcome measures such as the number of mutations detected, survival in the form of additional life years gained and QALYS are a suitable outcome for assessing the cost effectiveness of cancer genetic technology, they ignore the impact of testing upon families and society, and cost per mutation ignores the impact upon the individual.

Any future evaluations of cancer genetic services should, where possible, attempt to account for the impact of genetic services on the individual, the family and society (Hall *et al*, 1998). For individuals, the effects accounted for should include the value of information, the effects of choice and regret, and any unintended effects of health interventions. For families and social interaction, feelings of vulnerability, guilt, blame and continuous watching for early signs of disease should be considered. At a societal level, the issues to be addressed include discrimination, equality of access to technology, social pressure on choice and social expression of altruism and sympathy (Hall *et al*, 1998).

While expanding the range of outcomes to be considered is to be welcomed, this does raise implications for the form of analysis that can be performed. In the TRACE project (Brain *et al*, 2000; Cohen *et al*, in press) (which went beyond technology assessment to compare genetic assessment services with typical advice and surveillance from a breast clinic), cost–benefit, cost–utility and cost-effectiveness analysis were rejected in favour of a cost-consequences analysis. Cost–benefit was rejected on the grounds that the benefits and dis-benefits of the service were largely unknown and as such could not be valued. Cost–utility was disregarded due to the difficulty in measuring non-health benefits, dis-benefits and the psychological health benefits with relatively insensitive utility measures such as the EuroQol EQ-5D (EuroQol Group, 1990). Due to the wide range of likely outcomes of the genetic assessment of women at a high risk of breast cancer, it was felt that no single outcome could be selected for cost-effectiveness analysis.

Clearly, there is a need for further research on the psychosocial impact of genetic services upon the individual, family and society within a health economics context. There is also a need to assess the equity of access to genetic services in the light of Steel *et al*'s (1999) findings, which indicated that individuals from lower socio-economic backgrounds are under-represented as patients of genetics clinics.

#### Survival and QALYs

Although care is required in interpreting the results of some of the studies modelling life years, given the variations in parameter estimates used, they have consistently reported advantages for patients with an identified mutation if they receive presymptomatic surveillance and/or chemoprevention or surgical interventions. Vasen *et al* (1998) and Syngal *et al* (1999) have reported additional life years gained for patients at risk of developing colorectal cancer as a result of a HNPCC mutation. However, there is considerable divergence in their estimates of life years gained. Vasen *et al* (1998) estimated that colonoscopy every 2.5 years compared to no surveillance would provide 6.9 additional undiscounted life years for a male commencing surveillance at 25 years of age. Syngal *et al* (1998) found a gain of 13.5 undiscounted years for an individual opting for surveillance every 3 years from the age of 25. Despite utilising different mutation penetrance estimates, convergence was found in the estimated gains in life years for BRCA1/2-positive women opting for prophylactic surgery by Schrag *et al* (1997) and Grann *et al* (1998). Women opting for prophylactic mastectomy and oophorectomy at 30 years of age gained 6–7.6 undiscounted years over women having surveillance alone (see Table 3

**Table 3:** Life years gained for a 30-year-old woman with a BRCA1/2 mutation opting for immediate prophylactic surgery

	**Additional life years compared to surveillance alone**		
	**Scharg *et al* (1997)**	**Grann *et al* (1998)**	**Range**
*Low-risk penetrance*	BC 40%, OC 5%	BC 40%, OC 6%	
Oophorectomy	0.3	0.4	0.1
Mastectomy	2.9	2.9	0.0
Oophorectomy and mastectomy	3.2	3.3	0.1
			
*Medium-risk penetrance*	BC 60%, OC 20%	BC 56%, OC 16%	
Oophorectomy	1.0	0.9	0.1
Mastectomy	4.1	3.4	0.7
Oophorectomy and mastectomy	5.3	4.5	0.8
			
*High-risk penetrance*	BC 85%, OC 40%	BC 85%, OC 63%	
Oophorectomy	1.7	2.6	0.9
Mastectomy	5.3	2.8	2.5
Oophorectomy and mastectomy	7.6	6.0	1.6

BC=breast cancer penetrance, OC=ovarian cancer penetrance. All estimates of additional life years are undiscounted.

). Grann *et al* (2000, 2002) reported similar undiscounted findings to those in Table 3 for patients with moderate penetrance risks, an additional 0.8 years for a woman having an oophorectomy (Grann *et al*, 2000), 3.4 years (Grann *et al*, 2000) and 3.5 years (Grann *et al*, 2002) for mastectomy, 4.3 years (Grann *et al*, 2000) and 4.9 years (Grann *et al*, 2002) for a woman having both procedures. Divergence in results (Schrag *et al*, 1997; Grann *et al*, 1998, 2002; Syngal *et al*, 1998; Vasen *et al*, 1998; Wilson *et al*, 1999) stem from the parameter estimates used, in particular penetrance estimates, mortality rates and risk reductions as a result of surveillance and interventions. The modest gains in life years found in Schrag *et al*'s (2000) second study is predominantly a result of the cohort consisting of women at risk of developing genetic cancer for a second time. In stark contrast to Schrag *et al* (1997) and Grann *et al* (1998, 2000, 2002), Grann *et al*'s (1999) study looking at Ashkenazi women found very small gains in life expectancy for women opting for prophylactic surgery: 38 undiscounted days for a woman having both bilateral mastectomy and oophorectomy. This is due to the study being based on providing genetic testing and surveillance to all Ashkenazi women and not merely those at increased risk or having a positive mutation; Ashkenazi Jewish women also have lower mutation penetrance than women from other ethnic groups (Ford *et al*, 1995; Struewing *et al*, 1997).

QALYs gained from surveillance and interventions have been inconsistent. A woman with a moderate penetrance mutation in Grann *et al* (1998) gained 0.2 undiscounted QALYs from having both mastectomy and oophorectomy compared to 2.1 (Grann *et al*, 2000) and 2.6 (Grann *et al*, 2002) undiscounted QALYs in the revised models. This discrepancy is a result of the change in the QALY weightings and parameter estimates used in these studies. It has yet to be proven for genetic cancer that QALY utility scores can take full account of the psychosocial, physical and economic effects of testing. Van der Riet *et al* (1997) in their study modelling costs and outcomes for couples at risk of having a child with a monogenic disease state, ‘A traditional cost-effectiveness analysis, where costs per life years gained or QALY gained are measured, is not appropriate here since the different dimensions in which benefits can be expressed cannot be aggregated to a single summary outcome measure’ (Van der Riet *et al*, 1997, p 742).

In the case of HNPCC, both genetic testing accompanied by regular colonoscopy and polypectomy or having prophylactic surgery (Vasen *et al*, 1998) have been found to be cost effective in terms of life years saved. For women with BRCA1/2 mutations, both chemoprevention and prophylactic interventions have been found to be cost effective in terms of life years saved (Grann *et al*, 1998, 2000) and QALYs (Grann *et al*, 2000; Tengs and Berry, 2000). However, as is the case for surveillance alone, the benefits from prophylactic interventions have been found to diminish with older age or a delay in opting for the intervention (Schrag *et al*, 1997, 2000; Syngal *et al*, 1998; Vasen *et al*, 1998; Grann *et al*, 2000, 2002), and the long-term implications of chemoprevention drugs such as tamoxifen and raloxifene remain to be seen (Grann *et al*, 2000; Schrag *et al*, 2000). The data on QALYs indicates that surveillance (Syngal *et al*, 1998; Grann *et al*, 2000) and chemoprevention techniques (Grann *et al*, 2000, 2002) are favoured over prophylactic interventions.

It should be noted that all the modelling studies identified in this review have confined themselves to looking at identified mutations (BRCA1, BRCA2 or HNPCC). Families with an unidentified mutation are known to outnumber those with an identified mutation (Couch *et al*, 1997; Stoppa-Lyonnet *et al*, 1997; Eccles *et al*, 1998; Syngal *et al*, 1999).

### Financial cost of genetic services

#### Laboratory testing

There has been some variation in the results of the studies costing the provision of cancer genetic services. Estimated costs have ranged between €450 [£334^*^] and $1000 [£688] (Vasen *et al*, 1998) [£834^*^] (Brown and Kessler, 1995, 1996) for examining the hMLH1 and hMSH2 genes for HNPCC mutations (Debniak *et al*, 2000). Only one study considered the costs of testing for FAP, recoding costs of £207.12 [£234^*^] for testing a cancer-affected patient and £90.75 [£102^*^] for any subsequent member (Bapat *et al*, 1999). Familial adenomatous polyposis testing consisted of a protein truncation test of the APC gene if searching for the two most common mutations at APC codons 1061–1063 and 1309–1311 by heteroduplex analysis was unsuccessful. BRCA estimates have ranged between $70 [£48^*^] for a genetic test using extensive multiplex PCR amplification and two-dimensional electrophoresis (fragments coamplified) of BRCA1 (Van Orsouw *et al*, 1999), and $2400 for a full sequence analysis of the BRCA1 and BRCA2 genes by Myriad Genetics inc [£1591] (Grann *et al*, 2000; Lidereau *et al*, 2000). The disparity in costs is due to the purchase and maintenance costs of the respective equipment used, the number of exons searched and divergence in profit objectives between the public and private sectors.

#### Genetic counselling

The estimated cost of providing genetic counselling ranged between $129 [£89] (Cromwell *et al*, 1998) and $800 [£551] (Vasen *et al*, 1998). The most detailed micro-costing of a single genetics counselling session (breast, ovarian and colorectal cancer) was conducted at three clinics in Scotland (Wilson *et al*, 1999), providing a cost estimate of £86 [£94] to £192 [£210] to the NHS (costs in 1999/2000 £). The cost of counselling is particularly influenced by staff grade, preparation and counselling time.

### The value and utility of genetic services to patients

At present, information upon willingness to pay and the utility to patients of genetic testing and presymptomatic surveillance for familial cancer is sparse. The work of Chaliki *et al* (1995) has shown that if a token charge were implemented enthusiasm for a hypothetical offer of testing declined. There is currently no information upon the monetary value placed upon cancer genetic services by users and potential service users.

Wilson *et al* (1999) are the only ones to publish the results of a discrete choice conjoint analysis exercise with patients of cancer genetics services. This paper provides unique data upon the trade-offs patients are willing to make between service attributes and allows competing service configurations to be compared in terms of utility and cost utility. Although the majority of patients were found to prefer doctor-led counselling, they were willing to see a genetics nurse or genetics associate in favour of shorter waiting times and longer counselling sessions. In terms of patient utility, genetics nurses and associates were found to be cost effective compared to doctor-led counselling.

### Most efficient method of providing genetic services

#### Family history *vs* screening

Several studies have looked at the most effective way of utilising genetic testing. Initially, genetic testing was assessed as a population-screening tool (Brown and Kessler, 1995, 1996). Population testing for HNPCC was estimated to cost up to $2.6 million [£2.17 million^*^] per mutation detected (Brown and Kessler, 1995, 1996) and £114 240 [£138 280] per BRCA1 mutation detected (Eccles *et al*, 1998). Having established the high cost per mutation detected, clinicians and health economists have sought to target the use of testing to maximise mutation detection and minimise the expenditure (Tengs and Berry, 2000; Sevilla *et al*, 2002). Recent research has highlighted the efficiency of targeting high-risk cancer-affected patients and family members of those cancer-affected patients found to be gene positive (Cromwell *et al*, 1998; Eccles *et al*, 1998; Bapat *et al*, 1999; Heimdal *et al*, 1999; Debniak *et al*, 2000; Lidereau *et al*, 2000). There is some variation in the methods used to target cancer-affected patients and their families from family history alone to family history in conjunction with laboratory data such as immunohistochemistry and microsatellite instability. There is evidence of cost saving and prevention of unnecessary invasive surveillance techniques when strategic or targeted use is made of genetic testing, with little or no loss of sensitivity in mutation detection. Once a mutation is identified in a cancer-affected relative, any concerned family member can choose to be tested. Those with the mutation can be targeted for surveillance and those free of the mutation avoid the need for surveillance.

Widespread testing of entire ethnic groups was proposed by Grann *et al* (1999) and Heimdal *et al* (1999). Heimdal *et al* (1999) suspected that the prevalence of BRCA1 in some areas of Norway might justify considering testing the entire population. Grann *et al* (1999) advocated genetic testing for all Ashkenazi Jewish women for BRCA1/2. This is a result of mutation prevalence being estimated to be 2.5% in their model. As noted by Grann *et al* (1999), cost effectiveness would be improved if targeted testing based on family history could be conducted; however, frequently family history information is not available for these families as a result of small family size and the holocaust.

#### Cost effectiveness

Cost per BRCA1 mutation detected among members of high-risk families with a living cancer-affected relative or stored sample of their blood/DNA has been estimated to range between £74 [£90] (Eccles *et al*, 1998) and €6881.70 (£4328) (Sevilla *et al*, 2002). Estimates of the cost per life years saved by presymptomatic surveillance and/or prophylactic surgery ranged between £2100 [£2542] (Priority Areas Cancer Team, 1998) at a 6% discount rate for members of high-risk families, to $134 273 [£101 777] (Grann *et al*, 1999) at a 3% discount rate for surveillance of all Ashkenazi women. Cost per QALY were estimated to be $3500–4900 [£2314–3239] for high-risk women, $15 000 [£9916] for women at moderate risk, $34 000 [£22 477] for women at slightly increased risk and $1.6 million [£1.06 million] for women at population risk (Tengs and Berry, 2000). In addition to targeting high-risk families, cost effectiveness has been found to be influenced by the outcome measures used, estimated outcomes, mutation penetrance, mutation prevalence, the accuracy of testing, the cost of testing, the number of patients counselled per healthy mutation carrier, the frequency of clinical surveillance, health interventions employed and their efficacy, uptake and compliance, the age of the individual when having testing and/or prophylactic surgery, the proportion of early-to-late-stage cancer detected, prognosis of any cancer that develops and quality of life (Brown and Kessler, 1995, 1996; Grann *et al*, 1998, 1999, 2000; Priority Areas Cancer Team, 1998; Vasen *et al*, 1998; Heimdal *et al*, 1999; Tengs and Berry, 2000; Sevilla *et al*, 2002).

Wilson *et al* (1999) have begun to address the question set by Lerman (1997) on the most cost-effective way of delivering genetic services. In the case of counselling, there appears to be considerable advantage in utilising genetic associates and nurses rather than doctors and consultants to counsel in appropriate circumstances. Genetic associates and nurses are less expensive than consultants and were found to be acceptable to patients, particularly if this resulted in less travel to genetics counselling appointments and shorter waiting times. Obviously, there are many other service options to be considered and assessed.

In conclusion, modelling studies indicate that, for carriers of identified cancer-predisposing mutations surveillance, prophylactic and chemoprevention techniques extend survival. There is a need to build upon the findings of these studies and model survival and QALYs for high-risk patients with unidentified mutations and patients at moderate risk of developing genetic cancer. Both of these groups outnumber families with an identified mutation.

Depending upon the service protocols used, genetic testing has been estimated to cost between $70 and 2400 [£48^*^–1591] and genetic counselling between $129 and 800 [£89–551]. The technology of genetic testing and presymptomatic surveillance and interventions has been found to be cost effective in terms of mutations detected, survival and QALYs. Cost effectiveness was influenced by numerous factors, the main one being targeting genetic services for patients with a strong family history of cancer rather than screening the entire population.

In the past economic evaluations of cancer genetics services have concentrated on health outcomes and mutation identification. Future economic evaluation must go beyond health technology assessment and account for the impact of such services upon the individual, the family and society, and establish both the value of services to these groups and the most effective ways of delivering genetic services, offering counselling and information in addition to coordinated presymptomatic surveillance and genetic testing. Any future economic evaluations should also seek to avoid the deficiencies identified in preceding research relating to lack of detail upon costs and methods.
