# Supplementary material for: Cancer genetics services: a systematic review of the economic evidence and issues
Source: Br J Cancer. 2004 Apr 13;90(9):1697–703. doi: 10.1038/sj.bjc.6601792 (PMC2410279; doi:10.1038/sj.bjc.6601792)
Supplement: Supplementary Appendix [file 90-6601792x1.doc]

**Appendix: Summary of studies included in the systematic review**

| **Paper** | **Economic objectives** | **Methods** | **Results & conclusions** |
| --- | --- | --- | --- |
| Maher et al. (1993) Evaluation of molecular genetic diagnosis in the management of familial adenomatous polyposis coli: a population based study. *J Med Genet* **30**: 675 - 678 | Establish the monetary value of saved resources as a result of genetic testing for FAP. | **Evaluation:** Cost-benefit analysis.  **Perspective:** Third party payer.  **Sample, setting & methods:** 108 patients with a 50% chance of having inherited a FAP mutation (54 cancer affected & 64 at risk) were recruited for this hospital/lab based study. Costs taken from Walker et al. (1991). | Linkage analysis was found to be informative in most families. By discounting surveillance for 25 low risk family members 365 person years of surveillance were avoided. Based upon three yearly colonoscopy at £107 [£173] each time, this constitutes a saving of £13,000 [£20,560]. |

| **Paper** | **Economic objectives** | **Methods** | **Results & conclusions** |
| --- | --- | --- | --- |
| Chaliki et al. (1995) Women’s receptivity to testing for a genetic susceptibility to breast cancer. *Am J Public Health* **85**: 1133 - 1135 | Establish willingness to pay (WTP) >$25.00 for BRCA1 testing. | **Evaluation:** Cost-benefit analysis (willingness to pay).  **Perspective:** Third party payer.  **Sample, setting & methods:** 982 women, 484 awaiting mammography and 498 awaiting a gynaecology or obstetrics appointment were recruited to a questionnaire based survey. | 68% of mammography patients and 53% of obstetrics/gynaecology patients were willing to pay more than $25.00 [£21] for a hypothetical genetic test. Predictors of higher WTP amongst women included older age, currently having regular mammography, concerned about getting breast cancer, believed that mammography was effective at detecting early breast cancer, believed their risk to be high and believed that early breast cancer was curable. Women that were not pregnant compared to those that were, women whose mothers had had cancer compared to those that had not, and women accepting testing so they could take extra precautions rather than those seeking reassurance that their risk was low were also willing to pay more for testing. |

| **Paper** | **Economic objectives** | **Methods** | **Results & conclusions** |
| --- | --- | --- | --- |
| Brown ML, Kessler LG (1995) The use of gene tests to detect hereditary predisposition to cancer: Economic considerations. *J Natl Cancer Inst* **87**: 1131 – 1136  Brown ML, Kessler LG (1996) Use of gene tests to detect hereditary predisposition to cancer: What do we know about cost-effectiveness*? Int J Cancer* **69**: 55 - 57 | Explore the economic implications of conducting population-wide screening for HNPCC compared with restricted screening amongst members of families at high risk. | **Evaluation:** A Cost-consequences analysis.  **Perspective:** Third party payer.  **Sample, setting & methods:** The form of analysis was exploratory (modelling). Costs were based upon personal communications with a laboratory and estimates of cost efficient health resource use. Costs and estimates of gains in life years were discounted at 5%. | Although cost per case detected was higher for genetic testing than for conventional breast cancer screening, the cost per life year saved was more favourable due to the younger age of cancer onset. Population prevalence, the cost of genetic testing, life years gained, mutation penetrance and discount rate were found to affect cost-effectiveness substantially. The cost of the preventative intervention (endoscope and polypectomy) did not affect costs substantially. Only under very favourable assumptions such as a HNPCC prevalence of 50 cases per 10,000 people or more would population genetic testing, presymptomatic surveillance and intervention be cost-effective. Further information is required upon the determinants of the cots of genetic testing, counselling, the effectiveness of preventative interventions for high risk individuals, accuracy and costs of alternative methods of identifying high risk families. |

| **Paper** | **Economic objectives** | **Methods** | **Results & conclusions** |
| --- | --- | --- | --- |
| Heimdal et al. (1999) Costs and benefits of diagnosing familial breast cancer. *Dis Markers* **15**: 167 - 173 | Compare identification of BRCA1 carriers by family history followed by testing and the alternative of genetically testing all cases of detected breast and ovarian cancer. | **Evaluation:** Cost consequences analysis.  **Perspective:** Third party payer.  **Sample, setting & methods:** Modelling based upon findings at the Unit of Medical Genetics, Norwegian Radium Hospital, Norway. All costs were obtained from the Norwegian National Insurance Service. Costs also included the charges to patients for clinical examination and mammography. Costs per life year were not discounted. | The family history strategy was found to cost €753 [£558] per life years and €16,933 [£12,556] per cancer detected compared to €832 [£617] and €18,720 [£13,881] for the genetic testing of all breast cancer cases. Inherited breast cancer could be managed effectively for a cost of €750 – €1,600 [£556 - £1,186] per year for each patient. The prevalence of BRCA1 in some areas of Norway it was suggested may be sufficiently high to consider genetic testing of the entire population. |

| **Paper** | **Economic objectives** | **Methods** | **Results & conclusions** |
| --- | --- | --- | --- |
| Brain et al. (2000) Randomised trial of a specialist genetic assessment service for familial breast cancer. *J Natl Cancer Inst* **92**: 1345 - 1351  Cohen et al. (2003) Health Economics and Genetic Service Development - A cancer genetic example (TRACE). *Manuscript submitted for publication* | Compare the psychological impact and cost of providing typical advice and surveillance from a breast clinic with genetic assessment, genetic counselling and if appropriate genetic testing. | **Evaluation:** Cost consequences analysis.  **Perspective:** Societal.  **Sample, setting & methods:** Pragmatic randomised control trial of hospital based genetic testing and counselling. The cohort had 282 women in the control arm and 263 in the intervention. Costing was from a societal perspective, including forgone activities, child care and transport costs. NHS costs included capital, overheads, annuitised equipment costs (discount rate & lifespan not specified) and staff’s preparation time. All costs were presented in 1997/1998 £ and $ (Brain et al., 2000). | The only health outcome upon which a significant difference was found was greater knowledge of breast cancer for the intervention arm. Women in both groups experienced a significant reduction in anxiety and found attending the clinic to be highly satisfying. It should be noted that there was some evidence of dropout of patients being related to state and trait anxiety, perceived risk, age and cancer worry. 39 cancer affected relatives of the study cohort were visited, counselled and testing, resulting in 7 mutations being found. 6 cohort member and 13 relatives received cascade testing resulting in an incremental cost of £60.98 [£73] (Brain et al., 2000) per woman presenting to the genetic assessment arm. |

| **Paper** | **Economic objectives** | **Methods** | **Results & conclusions** |
| --- | --- | --- | --- |
| Eccles et al. (1998) BRCA1 mutations in Southern England. *Br J Cancer* **77**: 2199 - 2203 | Establish the most cost-effective method of identifying women for BRCA1 genetic testing. | **Evaluation:** A cost-effectiveness analysis.  **Perspective:** Third party payer.  **Sample, setting & methods:** Hospital/lab based study. Group 1 = 155 women that had developed breast cancer before they were 40. Group 2 = 45 women with bilateral breast cancer diagnosed after they were 39. Group 3 = 30 presymptomatic women with a family history of breast cancer, ovarian cancer or both. A micro costing excluding overhead costs appears to have been conducted. No costing methods were provided. | The most effective criteria for mutation detection was found to be early onset of cancer and a positive family history, with 6.5% of Group 1, 0% of Group 2 and 26.7% of group 3 found to have a BRCA1 mutation. Population testing was costly and impractical at an estimated £114,240 [£138,280] per mutation detected. By defining the mutation to be sought as is the case having tested a cancer affected relative as in group 1 (<£1,144 [£1,385] per detected mutation) and Group 3 (<£238 [£288] per detected mutation), costs can be kept relatively low. |

| **Paper** | **Economic objectives** | **Methods** | **Results & conclusions** |
| --- | --- | --- | --- |
| Lidereau et al. (2000) Major improvement in the efficacy of BRCA1 mutation screening using morphoclinical features of breast cancer. *Cancer Res* **60**: 1206 - 1210 | Establish the cost-effectiveness of detecting BRCA1 by mortphoclinical features. | **Evaluation:** A cost-effectiveness study.  **Perspective:** Third party payer.  **Sample, setting & methods:** Hospital/lab based study. 70 women that had contracted cancer by the age of 35 and for whom a tumour and lymphocyte DNA sample were available were recruited to the study. The samples were tested for the most discriminating features of BRCA1, tumour differentiation (TD3) and being oestrogen receptor negative (ER-). Genetic testing for BRCA1 was assumed to cost half of the cost of sequencing BRCA1/2. No details were provided upon costing sources. | Testing women with one or neither ER- and TD3 resulted in a 3.6% mutation detection rate, testing all 70 women in an 8.6% detection rate and testing TD3 and ER- women in a 28.6% detection rate. Extrapolating these results to the French population, some 1000 breast cancer cases would be expected annually in women of 35 years of age or younger. 200 cases would be expected to have tumours that are both ER- and TD3, and 800 cases would have either or neither of these features. This would result in cost-effectiveness ratios of $34,000 [£22,533] per mutation detected for women that had one or none of the clinical features, $14,000 [£9,278] if all 1000 women were tested and $4,200 [£2,784] if only women that were TD3 and ER- were tested. |

| **Paper** | **Economic objectives** | **Methods** | **Results & conclusions** |
| --- | --- | --- | --- |
| Debniak et al. (2000) Value of pedigree/clinical data, immunohistochemistry and microsatellite instability analysis in reducing the cost of determining hMLH1 and hMSH2 gene mutations in patients with colorectal cancer. *Eur J Cancer* **36**: 49 - 54 | Evaluate the significance of pedigree/clinical data (PCD), immunohistochemistry (IHC) and microsatellite instability (MI) analysis in the reduction of costs of constitutional hMLH1 and hMSH2 (HNPCC) gene mutation diagnosis in patients with colorectal cancer. | **Evaluation:** A cost-effectiveness study.  **Perspective:** Third party payer.  **Sample, setting & methods:** Hospital/lab based study. Group A = 43 consecutive sporadic late onset cases of colorectal cancer diagnosed at age 40 or above. Group B = 25 consecutive cases confirmed or suspected of having HNPCC. Costs were presented with no detail of their derivation. | No mutations were found amongst group A. All six mutations in group B were found when all 25 were tested by molecular genetic testing (sequencing) (PCD alone €3,750 [£2,781*] per mutation detected) or IHC and MI plus PDC (€1,767) [£1,310*] were used. Using PDC and IHC or MI, 83% of mutations (5 of 6) were found (€2,600 [£1,928*] MI + PCD or €880 [£653*] IHC + PCD). The exclusion of late onset sporadic tumours cases would result in the avoidance of sequencing in approximately 85% of cases. Sequencing should only be used in cases suspected or definitely having HNPCC based upon PCD when IHC and MI identify cases without alterations at the protein level. |

| **Paper** | **Economic objectives** | **Methods** | **Results & conclusions** |
| --- | --- | --- | --- |
| Sevilla et al. (2002) Testing for BRCA1 mutations: a cost-effectiveness analysis. *Eur J Hum Genet* **10**: 599 - 606 | Conduct a cost-effectiveness comparison between the main alternative strategies available for detecting BRCA1/2 mutations and the direct DNA sequencing method applied to the entire gene. | **Evaluation:** Cost-effectiveness.  **Perspective:** Third party payer.  **Sample, setting & methods:** A laboratory based study using a hypothetical cohort of 10,000 patients with a 15% probability of having a deleterious BRCA1 mutation. Direct DNA sequencing (DS) was compared with rapidly scanning part or the entire BRCA1 gene using 19 pre-screening strategies and conducting DS on any anomalies found. The 19 alternatives were based upon the pre-screening techniques of high performance liquid chromatography (DHPLC), single-strand conformation polymorphism (SSCP), denaturing gradient gel electrophoresis (DGGE), hetroduplex analysis (HA), fluorescent assisted mismatch analysis (FAMA) and the protein truncation test (PTT). Costs were derived by conducting a micro costing at 3 laboratories. The consumables, equipment and labour required for each test were measured and unit prices gained from the labs were applied to derive a cost estimate in 2002 €. Equipment was assumed to be used at full capacity and the labour force to be optimally productive. A 20% depreciation and 8% discount rate was used to calculate annual equipment costs. | DS, the gold standard was 100% sensitive, detected all 1500 mutations at a cost of €6881.7 [£4,328] per mutation. The costs of the alternative strategies tested were 30% - 90% lower than DS irrespective of how much use was made of the laboratory equipment. The most cost effective strategy was PTT on exon 11 and HA on the remaining 21 exons at a cost of €971.3 [£611] per mutation detected. As this strategy had a high false negative rate, detecting 1200 of 1500 mutations, 4 other strategies were found to be worth considering. PTT of exon 11 and DHPLC on the remaining 21 exons (€1038.5 [£653], 1410 of 1500 mutations), DHPLC of all 22 Exons (€1218.3 [£766], 1440 of 1500 mutations), FAMA exon 11 and DHPLC on the remaining 21 exons (€1563.6 [£983], 1470 of 1500 mutations) and FAMA alone of all 22 Exons (€4795.8 [£3,016], 1500 of 1500 mutations). As the firm that owns the patent on the BRCA1/2 genes only use DS, this may become the only testing procedure that can be used in future, preventing the use of more cost-effective strategies. |

| **Paper** | **Economic objectives** | **Methods** | **Results & conclusions** |
| --- | --- | --- | --- |
| Van Orsouw et al. (1999) A highly accurate, low cost test for BRCA1 mutations*. J Med Genet* **36**: 747 - 753 | Cost analysis of the sensitivity of extensive multiplex PCR and amplification followed by two dimensional electrophoresis. | **Evaluation:** Cost analysis.  **Perspective:** Third party payer.  **Sample, setting & methods:** A hospital/lab based study. DNA samples were taken from 60 women at high risk of having a BRCA1 mutation. A micro costing was conducted, but no details were provided of methods. | The 14 samples known to have a mutation were detected as were an additional 5 that had previously been unidentified. The cost of extensive multiplex PCR and amplification followed by two dimensional electrophoresis was estimated to be $70 [£48*] per test. The most expensive components of the manual test were labour and PRC enzymes whilst the cost of PCR primers was low. Labour and PCR enzyme costs would be significantly higher if each fragment had been separately magnified rather than co-amplified. It was reported that the cost of each test could be reduced to approximately $10 [£7*] per test with the use of commercially available PRC robotics and fluorescent imaging. |

| **Paper** | **Economic objectives** | **Methods** | **Results & conclusions** |
| --- | --- | --- | --- |
| Cromwell et al. (1998) Cost analysis of alternative approaches to colorectal screening in familial adenomatous polyposis. *Gastroenterology* **114**: 893 - 901 | Comparison of the cost of conventional sigmoidoscopy screening with genetic testing that begins with the cancer affected relative and genetic testing that commences with the at risk relative for FAP. | **Evaluation:** Cost minimisation.  **Perspective:** Third party payer.  **Sample, setting & methods:** Decision modelling. Costs were obtained from two third party payers. Genetic testing was costed at the commercial charge of a commercial laboratory. Costs were discount at 3%. | For a family with one 12 year old at risk, genetic testing beginning with a cancer affected relative followed by presymptomatic testing and surveillance for the child would cost $2,625 [£1,803] ($2,754 [£1,892] with counselling), $2,674 [£1,837] ($2,803 [£1,926] with counselling) when genetically testing the child first and $3,208 [£2,204] for conventional sigmoidoscopic screening. The cost savings increased as the number at risk increased. Testing the cancer affected relative in a family with 5 at risk members would save $5,515 [£3,789] over conventional sigmoidoscopic screening. Genetic testing can substantially reduce the cost of FAP surveillance and, when possible should start with a cancer affected family member. |

| **Paper** | **Economic objectives** | **Methods** | **Results & conclusions** |
| --- | --- | --- | --- |
| Bapat et al. (1999) Cost comparison of predictive genetic testing versus conventional clinical screening for familial adenomatous polyposis. *Gut* **44**: 698 - 703 | Conduct a cost comparison analysis of predictive genetic testing versus conventional clinical screening for individuals at risk of inheriting FAP, using the perspective of a third party payer. | **Evaluation:** Cost-minimisation.  **Perspective:** Third party payer.  **Sample, setting & methods:** Decision analysis model. Three strategies were considered. Strategy A comprised of 2 yearly sigmoidoscopy to age 35 years and every 3 to 5 years until 50 years for mutation carriers. Strategy B was annual sigmoidoscopy to the age of 50. The final strategy was clinical screening. Costs were calculated by conducting a micro-costing. Genetic testing costs included technologists’ labour, data interpretation and write-up by clinicians or scientists, genetics counselling, laboratory supplies and equipment and overheads. Supplies were valued at their replacement cost and included an estimate of wastage. Laboratory equipment was valued at replacement cost on an annualised basis with a 5% discount rate and an estimated working life of 5 years. 20% of total testing costs were allocated to overheads. Labour was costed at gross salary including benefits adjusted for holiday and sick leave. Labour costs included direct patient contact and indirect time such as paperwork. Clinical screening costs were measured and valued using identical methods. | For a family with one cancer affected member and 6 relatives, model A would cost £3,109 [£3,510*], model B £4,677 [£5,281*] and conventional clinical screening £5,019 [£5,667*]. For families with a pedigree structure suitable for linkage analysis, 99% accuracy and greater cost savings can be achieved. Substantial savings could be made by using a genetic rather than clinical strategy for patients at high risk of having a FAP mutation. In addition to the prevention of unnecessary expenditure, identification of non-mutation carriers will relieve anxiety, prevent the need for unnecessary clinical surveillance and allow resources to be directed at those patients at most risk of colorectal cancer. The authors did not feel the need to move from a biennial to annual sigmoidoscopy for FAP families based upon the results of their research. |

| **Paper** | **Economic objectives** | **Methods** | **Results & conclusions** |
| --- | --- | --- | --- |
| Wilson et al. (1999) *Assessing user preferences for, and costs of, genetic counselling for familial cancer risk in Scotland: a cost-utility analysis using conjoint analysis*. Chief Scientist Office | To elicit patient preferences for, and the costs of, alternative ways of providing counselling for familial cancer risk in Scotland. | **Evaluation:** Cost-utility.  **Perspective:** Societal.  **Sample, setting & methods:** A survey design was used, posting questionnaires to the homes of past attendees of four clinics in Scotland (N=538). Utility was measured using a discrete choice conjoint analysis questionnaire. The micro-costing was from a societal perspective including forgone activities, child care and transport costs to patients. NHS costs included capital, overheads, annuitised equipment costs (5yr life span & 6% discount rate) and staff’s preparation time. Costs were presented in 1999/2000 £. | Estimated average non-NHS costs per person ranged between £24.19 [£27] and £35.54 [£39]. The average cost of providing genetic counselling at Aberdeen, Edinburgh and Glasgow, including staff time (preparation etc), were £192 [£210], £86 [£94] and £136 [£149] respectively. Four service attributes were significant; staff seen at the appointment, waiting time till appointment, distance to appointment and duration of appointment. At each of the centres, maximum utility per pound was obtained by a scenario using nurse led counselling. In terms of patient utility, genetics nurses and associates were found to be cost effective compared to doctor led counselling. |

| **Paper** | **Economic objectives** | **Methods** | **Results & conclusions** |
| --- | --- | --- | --- |
| Vasen et al. (1998) A cost-effectiveness analysis of colorectal cancer screening of hereditary nonpolyposis colorectal carcinoma gene carriers. *Cancer* **82**: 1632 – 1637 | Analyse the cost-effectiveness of colorectal cancer surveillance of carriers of a mutated mismatch repair gene. | **Evaluation:** Cost consequences analysis.  **Perspective:** Third party payer.  **Sample, setting & methods:** Decision analysis model.  Costs were based upon the actual price in the Netherlands in 1997 and published studies (Wagner et al., 1996; Taplin et al., 1995). Costs were discounted at 5%. Life years were not discounted. | Surveillance with a 1 year interval and 2.5 year interval provide an additional 8.1years and 6.9 years respectively compared with no surveillance for a 25 year old male with a HNPCC mutation. Cost per life year saved for a 1 year interval was estimated to be $9,906 [£6,820] and $9,859 [£6,787] for a 2.5 year interval compared with the annual cost of $12,577 [£8,658] for a patient that had not received surveillance. |

| **Paper** | **Economic objectives** | **Methods** | **Results & conclusions** |
| --- | --- | --- | --- |
| Syngal et al. (1998) Benefits of colonoscopic surveillance and prophylactic colectomy in patients with hereditary nonpolyposis colorectal cancer mutations. *Ann Intern Med* **129**: 787 - 796 | To assess the life expectancy and quality adjusted life expectancy benefits derived from endoscopic surveillance and prophylactic colectomy for persons who carry a mutation associated with HNPCC. | **Evaluation:** Partial evaluation estimating life years and QALYs.  **Perspective:** Third party payer.  **Sample, setting & methods:** Markov model. The model was run for 60 cycles/years. Life years were discounted at 3%. | A 13.5 years gain in life expectancy was found for surveillance and polypectomy compared to no surveillance. Immediate prophylactic proctocolectomy and subtotal colectomy provided an incremental life expectancy of 15.6 years and 15.3 years respectively over no surveillance. Adjusting for quality adjusted life years (QALYs) resulted in surveillance and polypectomy providing most life years for patients. The decision to decline or have prophylactic surgery is very complex with personal preferences carrying great influence. |

| **Paper** | **Economic objectives** | **Methods** | **Results & conclusions** |
| --- | --- | --- | --- |
| Schrag et al. (1997) Decision analysis – effects of prophylactic mastectomy and oophorectomy on life expectancy among women with BRCA1 or BRCA2 mutations. *N Eng J Med* **336**: 1465 - 1471 | Compare prophylactic mastectomy and oophorectomy with no prophylactic surgery among women who carry mutations in the BRCA1 or BRCA2 gene. | **Evaluation:** Partial evaluation estimating life years.  **Perspective:** Third party payer.  **Sample, setting & methods:** Markov model. The model was run for 30 cycles/years. Life years were not discounted. | Having both mastectomy and oophorectomy at 30 years of age provided an additional 7.6 years for an individual with a high penetrance BRCA1/2 mutation (85% breast & 40% ovarian), 5.3 years for moderate penetrance (60% breast & 20% ovarian) and 3.2 years for low penetrance mutation (40% breast & 5% ovarian) compared to surveillance alone. |

| **Paper** | **Economic objectives** | **Methods** | **Results & conclusions** |
| --- | --- | --- | --- |
| Schrag et al. (2000) Life expectancy gains from cancer prevention strategies for women with breast cancer and BRCA1 or BRCA2 mutations. *JAMA* **283**: 617 - 624 | To examine the effects of tamoxifen therapy, bilateral oophorectomy, prophylactic contralateral mastectomy, and combinations of these strategies on life expectancy for women with unilateral breast cancer and a BRCA1 or BRCA2 mutation. | **Evaluation:** Partial evaluation estimating life years.  **Perspective:** Third party payer.  **Sample, setting & methods:** Markov model. The model was run for an unspecified number of cycles/years. Life years were not discounted. | Compared to surveillance alone, a 30 year old BRCA positive woman that has survived early stage breast cancer would gain 0.4 – 1.3 years from tamoxifen, 0.2 – 1.8 years from prophylactic oophorectomy, 0.6 – 2.1 years from prophylactic contralateral mastectomy and 0.8 – 4.4 years from all three procedures. Gains are positively related to the estimated penetrance of the mutations. Gains also decline in relation to older age at commencement of preventative strategy and poor prognosis from primary breast cancer (node positive). |

| **Paper** | **Economic objectives** | **Methods** | **Results & conclusions** |
| --- | --- | --- | --- |
| Grann et al. (1998) Decision analysis of prophylactic mastectomy and oophorectomy in BRCA1-positive or BRCA2-positive patients. *J Clin Oncol* **16**: 979 - 985 | Determine the survival, quality of life, and cost-effectiveness of prophylactic surgical procedures. | **Evaluation:** Cost consequences.  **Perspective:** Third party payer.  **Sample, setting & methods:** Markov model. The model was run for 50 cycles/years. Costs included in the model consisted of drug costs for chemotherapy, prophylactic mastectomy, breast surgery with reconstruction, prophylactic oophorectomy, gynaecologic surgery and treatment of metastatic disease. Costs took account of whether or not the organ at risk was surgically removed or not and all costs were converted to 1995 dollars. Costs were estimated from Medicare payments and part B payments to physicians. Costs and life years were discounted at 3%. | Oophorectomy and combined oophorectomy and mastectomy provided cost savings and additional life years for the three penetrance profiles set in the study. Oophorectomy and mastectomy provided an additional 3.3 to 6 years depending upon mutation penetrance. Having discounted QALYs in addition to costs, cost-effectiveness was only found for oophorectomy and mastectomy compared to surveillance alone when the maximum penetrance assumptions (85% breast & 63% ovarian cancer) were applied to the model (0.08 QALYs). Prophylactic interventions were found to be cost-effective compared to surveillance for life years saved but not quality adjusted life years. |

| **Paper** | **Economic objectives** | **Methods** | **Results & conclusions** |
| --- | --- | --- | --- |
| Grann et al. (1999) Benefits and Costs of Screening Ashkenazi Jewish Women for BRCA1 and BRCA2. *J Clin Oncol* **17**: 494 - 500 | Determine the survival benefits and cost-effectiveness of screening Ashkenazi Jewish women for the three specific BRCA1/2 gene mutations. | **Evaluation:** Cost-effectiveness.  **Perspective:** A public health/societal perspective was reported by the authors (costing is not entirely consistent with this perspective).  **Sample, setting & methods:** Markov model. The model was run for 50 cycles/years. Costs such as screening, surgery, treatments and drugs for chemotherapy were obtained from 1996 Fundamental Reference, Myriad Genetics and 1995 Medicare payments. All costs were expressed in 1995 US dollars and segregated in accordance with the patient opting for or against prophylactic surgery. Duration of survival was undiscounted, whilst a 3% discount rate was used for cost per life-year saved. | Testing and surveillance provided 6 incremental days and mastectomy and oophorectomy provided up to 38 incremental days over surveillance alone. Delaying oophorectomy had little impact upon survival; however this was not the case for women delaying mastectomy or both procedures. The median discounted incremental cost-effectiveness ratios per life-year saved was $20,717 [£15,703] for both mastectomy and oophorectomy, $29,970 [£22,717] for mastectomy, $72,790 [£55,174] for oophorectomy and $134,273 [£101,777] for surveillance alone. It may be reasonable to extend genetic testing, prophylactic surgery and surveillance to all Ashkenazi Jewish women for the BRCA1/2 mutations. |

| **Paper** | **Economic objectives** | **Methods** | **Results & conclusions** |
| --- | --- | --- | --- |
| Grann et al. (2000) Prevention with tamoxifen or other hormones versus prophylactic surgery in BRCA1/2-positive women: A decision analysis. *The Cancer Journal from Scientific America* **6**: 13 - 20 | Compare the outcomes of chemoprevention with tamoxifen, raloxifene, or oral contraceptives with the outcomes of prophylactic surgery among women with high risk BRCA1/2 mutations. | **Evaluation:** Cost consequences.  **Perspective:** Third party payer.  **Sample, setting & methods:** Markov model. The model was run for 50 cycles/years. Costs included were as per Grann et al. (1998) plus cost of dying with or without cancer and the cost of the chemopreventative drugs. All costs were in 1998 dollars. Cost sources were Medicare payments, part B payments to physicians, SEER-HCFA and the Pharmaceutical Fundamental Reference. Cost per life-year saved was discounted at 3%. | A 30 year old BRCA1/2 positive women’s survival could be prolonged by 0.8 years by bilateral oophorectomy, 3.4 years by bilateral mastectomy and in the case of both procedures 4.3 years in comparison with surveillance alone. Tamoxifen, raloxifene and oral contraceptives also provided incremental life years compared with surveillance, 1.6, 2.2 and 0.9 years respectively. Chemoprevention yielded more QALYs than prophylactic surgery. In comparison to surveillance alone, raloxifene added 3.2 QALYs, tamoxifen added 2.7 QALYs, oral contraceptives 1.4 QALYs, mastectomy 2.5 QALYs, oophorectomy 2.2 QALYs and mastectomy plus oophorectomy 2.1 QALYs. All chemoprevention and prophylactic surgeries were cost-effective compared to surveillance. The life years gained by tamoxifen were estimated to cost $1,879 [£1,242] per life year and $898 [£594] per QALY. Although surgery provided the most life years and cost reductions, quality of life issues made chemoprevention a more attractive option, particularly for younger women. |

| **Paper** | **Economic objectives** | **Methods** | **Results & conclusions** |
| --- | --- | --- | --- |
| Grann et al. (2002) Effect of prevention strategies on survival and quality adjusted survival of women with BRCA1/2 mutations: an updated decision analysis. *J Clin Oncol* **20**: 2520 - 2529 | Update findings regarding the effects of prophylactic surgery, chemoprevention, and surveillance on the survival and quality-adjusted survival of women who test positive for BRCA1/2 mutations. | **Evaluation:** Partial evaluation estimating life years and QALYs.  **Perspective:** Third party payer.  **Sample, setting & methods:** Markov model. The model was run for 70 cycles/years. Costs included Life years and QALYs were not discounted. | For a 30 year old BRCA1/2 positive women, compared to surveillance alone, survival could be prolonged by 0.1 years by having a child, 0.6 years by oral contraceptives, 1.8 years by tamoxifen, 2.6 years by oophorectomy, 3.5 years by mastectomy, 4.6 years by tamoxifen and oophorectomy, and 4.9 years by mastectomy and oophorectomy. The survival benefits declined as the age at which they were initiated increased, virtually disappearing after 60 years of age. The incremental QALYs recorded were 2.3 for oral contraceptives, 2.6 for mastectomy, 2.6 for mastectomy and oophorectomy, 2.8 for tamoxifen, 4.4 for oophorectomy and 6.3 for tamoxifen and oophorectomy. QALY benefits also declined with increased age at initiation of preventative strategy. |

| **Paper** | **Economic objectives** | **Methods** | **Results & conclusions** |
| --- | --- | --- | --- |
| Tengs et al. (1998) Testing for BRCA1 and BRCA2 breast-ovarian cancer susceptibility genes: A decision analysis. *Medl Decis Making* **18**: 365 - 375 | Evaluate the health implications of testing for mutations in the BRCA1 and BRCA2 breast-ovarian cancer susceptibility genes. | **Evaluation:** Partial evaluation estimating life years and QALYs.  **Perspective:** Third party payer.  **Sample, setting & methods:** Markov model. The model was run for an unspecified number of cycles/years. Life years and QALYs were not discounted. | In comparison with not having genetic testing, mastectomy and oophorectomy at 30 years of age provide the maximum survival regardless of whether or not a woman had a BRCA1/2 mutation. A test with 100% sensitivity and specificity was found to yielded 50% more QALYs than a test with 80% sensitivity and 99% specificity. Allowing for QALYs, the optimal intervention depends upon the probability of having a mutation. For a probability of 0.0 - 0.12 no intervention was recommended; between 0.12 – 0.88 oophorectomy and for 0.88 or above both oophorectomy and mastectomy were recommended. The ideal candidate for testing was at moderate to high risk of having a mutation with no more than moderate concern about the impact of prophylactic surgery upon their quality of life. Testing was not recommended for women at population risk as it is unlikely to substantially improve their survival or quality of life. |

| **Paper** | **Economic objectives** | **Methods** | **Results & conclusions** |
| --- | --- | --- | --- |
| Tengs TO, Berry DA (2000) The cost effectiveness of testing for the BRCA1 and BRCA2 breast-ovarian cancer susceptibility gene. *Disease Management and Clinical Outcomes* **2**: 15 - 24 | What is the cost-effectiveness of testing for mutations in the BRCA1 and BRCA2 genes? Over what range of hereditary risk, if any, is testing cost-effective? How sensitive are the study findings to the specific parameter choices employed. | **Evaluation:** Cost-effectiveness.  **Perspective:** Societal (costing was more in keeping with the perspective of a third party payer).  **Sample, setting & methods:** Markov model. The model was run for an unspecified number of cycles/years. Costing was from a third party payer’s perspective. Model parameters and costs were obtained from peer review journals, government data sets (SEER), company websites (Myriad) and a survey of breast cancer experts. Cost estimates were updated to 1998 U.S. dollars using the medical consumer price index. Costs, life years and QALYs were discounted at 3%. | For women at population risk the cost-effectiveness ratio was $1.6 million [£1.06 million] per QALY gained. The cost per QALY gained for women at slightly increased risk was $34,000 [£22,477], $15,000 [£9,916] for women at moderate risk, and $3,500 to $4,900 [£2,314 - £3,239] for high risk women. The costs and QALYs of testing were dependent upon the prophylactic intervention employed in light of test results. For gene positive women the model revealed in all cases that oophorectomy at 30 or even at 50 was the best option. |

| **Paper** | **Economic objectives** | **Methods** | **Results & conclusions** |
| --- | --- | --- | --- |
| Lerman C (1997) Translational behavioural research in cancer genetics. *Preventative Medicine* **26**: S65-S69 | Summarise the results of National Cancer Institute Workshop with behavioural scientists to identify priority research areas. | Review article (not systematic). | Brown and Kessler (1995; 1996) were criticised for not consider quality of life outcomes in their cost-effectiveness analysis, and failing to account fully for the cost of pre-test education and post-test counselling. As was suggested by Brown and Kessler (1995; 1996), Lerman felt that economic analysis to identify the most cost-effective method of delivering genetic testing and counselling services are required. Three main areas that would impact on cost-effectiveness were identified. Firstly, some protocols have been developed from Huntington’s Disease protocols. The usefulness of protocols with extensive pre-test assessment to identify depression and suicidal potential in the context of cancer was questioned. Secondly, does genetic testing for cancer require multiple visits following test results as is recommended for Huntington’s Disease? Finally, a comparison of the cost-effectiveness of delivering genetic services by different clinicians and in different settings was called for. |

| **Paper** | **Economic objectives** | **Methods** | **Results & conclusions** |
| --- | --- | --- | --- |
| Peters JA, Biesecker BB (1997) Genetic counselling and hereditary cancer. *Cancer* Supplement **80**: 576 - 586 | Summary of a Workshop on genetic counselling and hereditary cancer presented at the American Cancer Society Workshop on Heritable Cancer Syndromes and Genetic Testing. | Review article (not systematic). | Based upon the literature they had reviewed they concluded that cost effectiveness would need to be established independently for different populations. |

| **Paper** | **Economic objectives** | **Methods** | **Results & conclusions** |
| --- | --- | --- | --- |
| Priority Areas Cancer Team/ Genetics Sub-committee of the Scottish Cancer Co-ordinating and Advisory Committee (1998) *Cancer Genetics Services in Scotland*. Scottish Office | Assessment of the costs and benefits of establishing screening programmes for individuals with a significant family history of breast, ovarian or colorectal cancer. | A review (not systematic). No details are provided on the sources of the costs used. | Six costs and four benefits of establishing such a program were identified. The costs were education and training for primary care staff and health information leaflets for patients, genetic clinics, raised anxiety levels in patients, management protocols, risks associated with surveillance methods and treatment. The benefits were information that reassures patients, savings life years, improved quality of life and avoided treatment. Cost per life years saved were estimated to be £2,100 [£2,542*] for breast, £3,000 [£3,631*] for colorectal and £7,900 - £15,800 [£9,562* - £19,125*] for ovarian cancer. The report concluded that presymptomatic surveillance could be cost effective for breast and colorectal cancer and that surveillance programs should be implemented. |

| **Paper** | **Economic objectives** | **Methods** | **Results & conclusions** |
| --- | --- | --- | --- |
| Hall et al. (1998) Taking a count: the evaluation of genetic testing. *Aust N Z J Public Health* **22**: 754 - 758 | Reviewed the issues relating to the economic evaluation of genetic testing from a social perspective. | A review (not systematic). | The immediate outcome of genetic testing is information and not a change in health. In the past evaluations have confined themselves to positive health effects and those utilising a social perspective have confined themselves to the total effects on individuals. Implementing such approaches to evaluating new genetic tests, in particular multifactorial disorders, late onset and less severe conditions will exclude significant effects for the individual, family and society. For individuals the effects accounted for should include the value of information, the effects of choice and regret, and any unintended effects of health interventions. For families and social interaction, feelings of vulnerability, guilt, blame and continuous watching for early signs of disease should be considered. At a societal level the issues to be addressed include discrimination, equality of access to technology, social pressure on choice and social expression of altruism and sympathy. The commercial sector and the availability of the technology will serve to encourage demand for widespread testing. Enthusiasm for evidence based medicine and policy will call for the economic evaluation of new genetic testing and technology, however, the array of benefits and dis-benefits are not easily measured and valued by current methods. |

| **Paper** | **Economic objectives** | **Methods** | **Results & conclusions** |
| --- | --- | --- | --- |
| Steel et al. (1999) Ethical, social and economic issues in familial breast cancer: A compilation of views from the E.C. Biomed II Demonstration project. *Dis Markers* **15**: 125 - 131 | Collating views gathered upon the ethical, social and economic issues raised as a result of genetic breast cancer. | A Survey of 12 cancer genetics clinics in Europe. | Although breast cancer is known to be more common in women of a high social class, this group were disproportionately prominent among the patients of seven out of the twelve European familial breast cancer clinics surveyed. In Scotland high numbers of patients were found to be graduates and health care workers. Whilst this phenomenon is not uncommon in health promotion programs it is reportedly more extreme for familial cancer. In Edinburgh patients categorised as blunters on the Miller Behavioural Scale were less likely to be referred by their GP if they were from a deprived group. |

| **Paper** | **Economic objectives** | **Methods** | **Results & conclusions** |
| --- | --- | --- | --- |
| Edwards RT (2001) Steering a course around the genetics iceberg. *J Public Health Med* **23**: 3 - 4 | Explore some of the divergent concerns about the implications of genetic testing for the United States and the United Kingdom. Asks how the benefits of information about the risk of cancer and other diseases are to be measured and valued. | A Review/debate article (not systematic). | Genetic testing has the potential to create an underclass, unable to afford health insurance and gain employment. In the UK concerns centre around demand for genetic testing and counselling. In the face of calls for expansion, services need to be evaluated so they can be targeted at those individuals that can benefit most. For breast and ovarian cancer it is relatively easy to calculate the benefits of life expectancy and quality of life resulting from early diagnosis and treatment for mutation carriers. How should the benefits and value of, information on risk status and counselling be measured? Does the commercial price of testing reflect the value placed upon NHS testing and what added value is contributed by NHS counselling? The NHS faces three potential models for the development of genetic services: 1) human rights, offering citizens information upon which to base decisions about their health, 2) utilitarian, aiming to reduce the prevalence of disease or disability in society, 3) clinical ethics, genetic testing should be developed and offered only where there is a corresponding treatment available. |
